# The Role of Glutamate and Blood–Brain Barrier Disruption as a Mechanistic Link between Epilepsy and Depression

**DOI:** 10.3390/cells13141228

**Published:** 2024-07-21

**Authors:** Benjamin F. Gruenbaum, Antonia Schonwald, Matthew Boyko, Alexander Zlotnik

**Affiliations:** 1 Department of Anesthesiology and Perioperative Medicine, Mayo Clinic, Jacksonville, FL 32224, USA; 2 New York Medical College, Valhalla, NY 10595, USA; aschonwa@student.nymc.edu; 3 Department of Anesthesiology and Critical Care, Soroka University Medical Center, Ben-Gurion University of the Negev, Beer-Sheva 84101, Israel; matthewboykoresearch@gmail.com (M.B.); alexander.zlotnik.71@gmail.com (A.Z.)

**Keywords:** blood–brain barrier, depression, epilepsy, glutamate, neurodegeneration

## Abstract

Epilepsy is associated with substantial neuropsychiatric impairments that persist long after the onset of the condition, significantly impacting quality of life. The goal of this review was to uncover how the pathological consequences of epilepsy, such as excessive glutamate release and a disrupted blood–brain barrier (BBB), contribute to the emergence of neuropsychiatric disorders. We hypothesize that epilepsy induces a dysfunctional BBB through hyperexcitation, which then further amplifies post-ictal glutamate levels and, thus, triggers neurodegenerative and neuropsychiatric processes. This review identifies the determinants of glutamate concentration levels in the brain and explores potential therapeutic interventions that restore BBB integrity. Our focus on therapeutic BBB restoration is guided by the premise that it may improve glutamate regulation, consequently mitigating the neurotoxicity that contributes to the onset of neuropsychiatric symptoms.

## 1. Introduction

Epilepsy affects approximately 50 million people worldwide and is defined as an unprovoked occurrence of seizures [1]. Without appropriate intervention, epileptic seizures recur, subsequently causing repeated insults to the brain. While the neuropathological predisposition to seizures remains under extensive investigation, seizures themselves arise from hypersynchronous and excessive neuronal excitation [2]. Hyperexcitability is often driven by high levels of glutamate but can also be achieved through a loss of inhibition through gamma-aminobutyric acid (GABA) [2]. The state of hyperexcitability leads to a wide range of symptoms, from brief lapses of attention seen in absence seizures to long-lasting convulsions and loss of consciousness seen in status epilepticus (SE) [3].

While seizures are the primary manifestation of epilepsy, accumulating evidence points to a complex interplay between epilepsy and various neuropsychiatric disorders. Individuals affected by epilepsy exhibit a significantly heightened risk of developing a neuropsychiatric disorder compared to the general population [4,5,6]. The relationship is believed to be bidirectional in instances of mood disorders and anxiety [6,7,8]. A total of 30% of epilepsy patients experience symptoms of depression and anxiety, which have a significant impact on their quality of life and treatment outcomes [5,6]. Furthermore, cognitive deficits, such as memory impairment and attentional difficulties, are prevalent among epilepsy patients, even in the absence of frequent seizures [9,10]. Moreover, the chronic stress associated with living with epilepsy, including social stigma, medication side effects, and the fear of seizures, can exacerbate psychiatric symptoms and further complicate management strategies [11].

Despite growing evidence of the intricate relationship between epilepsy and neuropsychiatric disorders, many aspects of this association remain poorly understood. Emerging research indicates that common pathophysiological processes, including alterations in neurotransmitter release, neuroinflammation, and structural brain changes, may contribute to the development of both conditions [12,13]. This review focuses on understanding how the disruption of the blood–brain barrier (BBB), as a consequence of excessive glutamate release in epilepsy, may trigger and exacerbate neuropsychiatric disorders.

Consisting of tightly joined endothelial cells along with a basement membrane, pericytes, and astrocytic end-feet, the BBB relies on tight and adherens junctions between the endothelial cells to restrict paracellular transport [14]. Additionally, transport proteins and efflux pumps enable the movement of nutrients and assist with disposing of harmful substances [15]. Microglia also contribute to the integrity of the BBB by providing protection in the brain against toxins and pathogens [15].

Various pathological processes, such as inflammation, oxidative stress, and hyperexcitability, can compromise BBB integrity [16,17]. In epilepsy, excessive glutamate evokes endothelial dysfunction and a breakdown of the extracellular matrix, which drive the failure of BBB [17]. This disruption facilitates the entry of blood-borne molecules, immune cells, and cytokines into the brain parenchyma, triggering inflammatory responses and further neuronal damage [18]. This dysfunction also contributes to an increase in glutamate levels, which further amplifies the downstream neurodegenerative mechanisms, manifested through cognitive and behavioral deficits associated with epilepsy.

**Figure 1:**
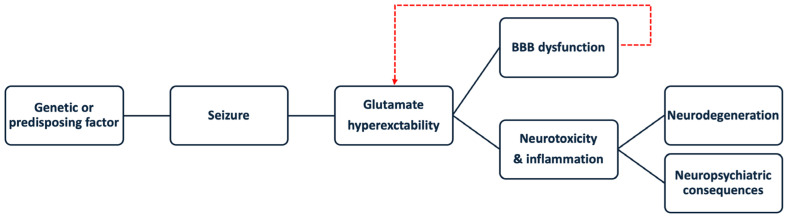
Role of excessive glutamate and blood–brain barrier (BBB) dysfunction in the mechanisms that contribute to neuropsychiatric consequences.

Elucidating the underlying pathophysiology and identifying effective strategies for intervention is crucial for improving the care of individuals afflicted by epilepsy. Figure 1 depicts the proposed mechanism of pathogenesis covered in this review. We aim to provide an overview of the current literature that may instruct future clinical practice and research.

## 2. Interplay of Glutamate in Epilepsy and Neuropsychiatric Disorders

Glutamate is a well-known excitatory neurotransmitter in the brain. It plays a pivotal role in long-term potentiation and synaptic plasticity, which are the physiological building blocks of learning and memory [19]. Glutamate has also been a focal point of epilepsy research due to its extensive involvement in seizures. Hypersynchronous and excessive releases of glutamate are triggered by the onset of seizures [20].

Evidence from animal models and human studies during SE highlights the involvement of glutamate in seizures and epileptogenesis [20,21,22]. Increased levels of glutamate in the extracellular space during seizures often lead to excitotoxic damage [20]. Prolonged seizures increase the expression of glutamate receptors, such as the N-methyl-D-aspartate (NMDA) receptor, further contributing to the development of epilepsy [20]. They also decrease the number of glutamate uptake transporters and contribute to the internalization of the GABA receptors in neurons and glial cells, which reduces inhibition and promotes hyperexcitability [23]. Consequently, drugs targeting GABAergic neurotransmission often fail to suppress seizures associated with sustained SE in experimental settings, whereas treatment with NMDA receptor antagonists alongside GABA agonists and other agents can effectively attenuate seizures [20]. NMDA receptor antagonists such as MK-801 and ketamine have been shown to suppress seizures [24]. They also offer neuroprotection after prolonged SE through the preservation of cognition and decreased anxiety [25], emphasizing the therapeutic importance of glutamatergic modulation as a preventor of seizure-induced damage. Hence, targeting the glutamatergic system with NMDA receptor antagonists may prevent long-term pathological alterations in synaptic connectivity and stop the progression of epileptogenesis [20].

There is accumulating evidence that points to a common glutamate-related pathology that underlies epilepsy and other central nervous system (CNS) disorders, such as depression, which is often comorbid with epilepsy [20,26]. Glutamate has been extensively studied in the context of learning, memory, depression, and anxiety [19,27,28,29,30]. Mood disorders and anxiety have traditionally been targeted by monoamines, benzodiazepines, or cannabidiol [31,32,33]. However, there is growing interest in the role of glutamate in the etiology of these disorders, in addition to targeting the glutamatergic system as a form of therapy [30,33,34,35,36]. Medications aimed at modulating glutamate release or antagonizing NMDA receptors are already increasingly utilized in the treatment of anxiety disorders [32,33,37]. While therapies targeting glutamatergic neurotransmission exist, many encounter challenges due to adverse effects [20]. Advancing our current understanding of these systems is crucial to identifying new therapeutic targets that directly and indirectly modulate glutamatergic signaling. Hopefully, targeting these pathways will yield better therapeutic management of both epilepsy and its neuropsychiatric comorbidities.

## 3. Glutamate Dysregulation in Epilepsy

Neuronal depolarization causes a calcium-dependent release of glutamate into the synaptic cleft [20]. Glutamate then exerts its effects by binding to several ionotropic and metabotropic receptors. Of note are the fast ionotropic α-amino-3-hydroxy-5-methyl-4-isoxazole propionic acid (AMPA) receptors and the slow metabotropic NMDA receptors that both evoke excitatory postsynaptic potentials [20,38]. The strength and frequency of these potentials orchestrate synaptic plasticity and normally contribute to the underlying mechanisms of learning and memory [19].

However, the modulation of these receptor subtypes can also contribute to aberrant neuronal activity, as neurons can strengthen or weaken their response to excitatory inputs. Past synaptic events and the underlying neural circuitry govern synaptic strength [20,23]. On a cellular level, synaptic strength is predominantly dependent on the number, localization, and subunit composition of the AMPA receptors or the gatekeepers of postsynaptic plasticity [23,39]. Changes that occur on a cellular level ultimately contribute to the remodeling of the synapse and, consequently, the wiring of the neurocircuit.

In epilepsy, such cellular changes can promote destabilization that results in hyperexcitability. Destabilization changes the ease with which a postsynaptic neuron can be depolarized by a presynaptic input [40]. Under physiological circumstances, neurons uphold a certain threshold that ensures synaptic homeostasis, as seen in long-term potentiation [20,41]. However, in epilepsy, dyssynchronous network activity may alter the circuit’s threshold, causing changes in excitatory responses, which then contribute to the rewiring of the neuronal network [20]. This rewiring further induces hyperexcitable states and forms neuronal connectivity that contributes to both ictogenesis and epileptogenesis [20]. These hyperexcitable and glutamatergic-driven states then trigger other pathological mechanisms downstream, such as neuronal cell death, inflammation, neurodegeneration, stress, and loss of synaptic integrity.

However, through a mechanism resembling a positive feedback loop, glutamate concentrations can be further amplified in the body through the disruption of the BBB [17,42,43]. We hypothesize that this glutamate-driven insult contributes to increased glutamate concentrations during post-ictal periods, further increasing glutamatergic-associated neurotoxicity that intensifies neuropsychiatric symptoms in epilepsy.

## 4. BBB Dysfunction in Epilepsy

The destruction of the BBB in epilepsy is a complex issue and has resulted in many conflicting studies from the neuroscience community. However, recent evidence suggests that the damage to BBB increases seizure incidence and occurrence, hence actively promoting epileptogenesis [16,18,42,43].

The pathogenesis points to an acute phase and a chronic phase of BBB insult in epilepsy. During the acute phase, there is a transient and focal increase in the permeability of the BBB. This is especially evident during severe ictal activity such as that seen in SE [16]. The chronic phase occurs after repeated insults caused by the hyperexcitable state of seizures [17]. Excessive glutamate causes an increased release of cytokines, heightened production of free radicals, and modified function of pericytes, which contribute to the extravasation of serum components, such as albumin [44]. The entry of blood-borne substances into a previously immuno-privileged brain triggers a series of immune responses mediated by the resident glial cells [12]. The inflammation is driven even further through blood-borne leukocytes migrating into the brain parenchyma and further disrupting extracellular homeostasis. Histologically, this phase is also characterized by a reduction in tight junctions and increased activation of matrix metalloproteinases. Both processes drive the destruction of the extracellular matrix and further compromise the integrity of the BBB, which increases permeability [17]. Increased transport of ions across the BBB and altered astrocytic potassium buffering lowers the threshold for spreading depolarization and causes neuronal hyperexcitability [44]. The concurrent dysfunction of glial cells and hyperexcitability of neurons is associated with the occurrence of epilepsy, cognitive decline, and behavioral abnormalities following BBB dysfunction.

The genesis of a positive feedback loop through which a damaged BBB continues to injure an already injured brain has one neurotransmitter that is likely responsible: glutamate. As one of the most abundant free amino acids in the brain, at a concentration of 10,000–12,000 μM/kg in the brain parenchyma, glutamate is kept in its respective compartments through the active transport of the BBB [33]. A healthy BBB heavily regulates the transfer of glutamate between the intraparenchymal and blood compartments. The extracellular space contains only 1–10 μM/L, while the plasma and whole blood are 50–100 μM/L and 150–300 μM/L, respectively [33].

The BBB acts as a natural regulator of any pathological increases in glutamate in the cerebrospinal fluid (CSF) and the extracellular fluid (ECF) in a healthy brain. Through its structural integrity and membrane transporters, it lowers the availability of glutamate by removing it from the brain into the blood [45]. In epilepsy, the chronic changes to the integrity of BBB compromise its ability to clear glutamate that is released excessively during seizures, as well as the neurotoxic events that follow them.

The added disruption of glutamate homeostasis, on top of heightened excitability due to ion disbalances, led us to the following hypothesis: the BBB acts as a major modulator of glutamate in CSF and ECF in epilepsy and contributes to the neurodegenerative processes that ultimately lead to mental disorders by promoting hyperexcitability.

## 5. Neurotoxicity

There are several mechanisms that are attributed to glutamatergic neurotoxicity, either because of epilepsy or a dysregulated BBB. This review focuses on exploring the mechanisms of neuronal cell death, neuroinflammation, stress, and loss of synaptic integrity.

### 5.1. Neuronal Cell Death

The extent to which seizures and epilepsy contribute to neuronal cell death is still heavily debated. However, severe and recurrent seizures, such as the ones seen in SE, are known to induce neuronal cell loss [46,47]. Given that SE often precipitates the onset of epilepsy, one can argue that neuronal loss facilitates epileptogenesis. Perhaps through neuronal cell death, seizures propagate through circuits that contribute to further hyperexcitation, ridding the brain of any regulation in firing. Current research emphasizes mechanisms of cell death that extend beyond conventional concepts of necrosis and apoptosis, encompassing autophagy, phagoptosis, necroptosis, and pyroptosis [46].

Furthermore, the process of neuronal death is known to be triggered by extensive glutamate stimulation, which paradoxically releases even more glutamate. In epilepsy, there is an overactivation of the glutamate receptors that patrol intracellular calcium [47]. Upon stimulation, there is an increased release of calcium into the cytoplasm, which triggers proteolytic enzymes and apoptosis. Additionally, it also causes a further release of glutamate, propagating a hyperexcitable state and damaging the BBB. As discussed earlier, the BBB further contributes to glutamate release by creating an ionic imbalance that favors excitation. Blocking glutamate release or binding (through NMDA antagonists) has been found to be neuroprotective [47].

The process of cell death follows immediately after a seizure if the excitation is large enough to warrant excitotoxicity [46]. The first minutes following the insult are characterized by significant neuronal loss followed by an additional release of glutamate into the ECF [33]. Depending on the origin and level of cortical activation of the seizure, apoptosis may be ongoing for several days [33]. Given the acuity of this mechanism of neurotoxicity, it is unlikely that neuronal cell death fully accounts for the occurrence of neuropsychiatric disorders in epilepsy, which manifest chronically.

### 5.2. Neuroinflammation

As previously described, hyperexcitability during seizures is one of the key contributors to neuroinflammation. This inflammation can be further exacerbated with a compromised BBB, which allows access to unwanted inflammatory cytokines and inflammatory cells that contribute to the destruction of the neuronal tissue through additional excitotoxicity.

Glutamate-driven inflammation may contribute to the development of mood disorders, as patients with mood disorders have been observed to have increased inflammation [48]. Furthermore, treatment with anti-inflammatory treatments, such as nonsteroidal anti-inflammatory drugs, has been shown to reduce depressive symptoms [49]. Neuroinflammation has also been associated with an altered metabolism of glutamate, which contributes to symptoms like anhedonia and psychomotor slowing [48].

While neuroinflammation is a tempting explanation for the neuropsychiatric disorders associated with epilepsy, it raises the question of why there is no neuropsychiatric presentation in other cases of neuronal inflammation, such as encephalitis or meningitis. However, research on this topic is limited and has only recently been gaining more attention [50]. Furthermore, these inflammatory processes are more acute than the chronic persistence of epilepsy. It may be that chronic levels of inflammation that are driven by BBB dysfunction contribute to comorbidity with psychiatric disorders. However, this hypothesis is understudied and warrants further investigation.

### 5.3. Stress

Stress and glutamate have been studied extensively in the context of anxiety and mood. Pharmacological targeting of the NMDA receptor through ketamine has been shown to be effective in treating anxiety and depression [51,52]. Increased glutamate in the anterior cingulate cortex has also been shown to induce a state of panic in healthy individuals [53]. However, more research is needed to further elucidate the association between glutamate and stress.

### 5.4. Loss of Synaptic Integrity

Astrocytes promote communication between neurons and Schwann cells in the peripheral nervous system through the release of adenosine triphosphate (ATP) [54]. In the CNS, the release of ATP can modulate the synapse. Glutamate can prompt this release and cause homosynaptic or heterosynaptic suppression, further integrating itself in synaptic modulation outside of the AMPA or NMDA receptors [55].

## 6. Neurodegeneration

There is accumulating evidence that demonstrates an association between glutamate neurotoxicity and neurodegeneration in epilepsy [56]. As previously mentioned, excessive glutamate concentrations can trigger neuronal degeneration and dysfunction as a consequence of neurotoxicity. Recent research has also explored how mood and anxiety disorders might be targeted through the glutamatergic system [6,35].

In acute settings, elevated extracellular glutamate concentrations can trigger excitotoxicity by activating ionotropic glutamate receptors, particularly following an acute brain insult such as a seizure. Chronic exposure to excessive glutamate has been implicated in numerous neurodegenerative disorders, such as Alzheimer’s disease, amyotrophic lateral sclerosis, and Huntington’s disease [33]. In such cases, prolonged exposure to elevated glutamate concentrations may slowly lead to cellular death or impaired neuroplasticity [56]. We postulate that therapeutic approaches targeting these conditions may operate by specifically reinstating glutamatergic homeostasis through the augmentation of glutamate uptake and the modulation of extracellular glutamate levels. The cascade from glutamate neurotoxicity to neurodegeneration, thus, presents novel avenues for the development of treatments for both acute and chronic neurological conditions.

## 7. Genetic Factors Influencing BBB Permeability, Seizure Susceptibility, and Glutamate Sensitivity

Genetic factors play a crucial role in determining the integrity of the BBB, susceptibility to seizures, and sensitivity to glutamate, thereby influencing the complex interplay between epilepsy and depression. Several genes have been identified that impact BBB permeability. For example, the gene encoding claudin-5, a tight junction protein, is critical for maintaining BBB integrity [57,58]. Mutations or polymorphisms in this gene can compromise BBB function, leading to increased permeability and subsequent neuroinflammation, which is a common feature in both epilepsy and depression [59]. Additionally, genes such as MMP-9, which encodes matrix metalloproteinase-9, can degrade extracellular matrix components and disrupt the BBB during epileptic events, further exacerbating neuronal injury and inflammation [17,60,61].

In terms of seizure susceptibility, genetic variations in ion channel genes, such as SCN1A, which encodes the alpha subunit of the voltage-gated sodium channel, have been linked to various forms of epilepsy [62,63]. These mutations can alter neuronal excitability and enhance the propensity for seizure generation. Similarly, genetic variations in glutamate receptors, like GRIN2A, which encodes a subunit of the NMDA receptor, can modify glutamate signaling pathways [64,65]. Abnormalities in these receptors can lead to glutamate hyperexcitability, a hallmark of both seizure activity and excitotoxicity that contributes to neuropsychiatric disorders, including depression.

Furthermore, genetic factors affecting glutamate metabolism, such as variations in the SLC1A1 gene encoding the excitatory amino acid transporter 3 (EAAT3), can influence synaptic glutamate levels [66]. The reduced functioning of EAAT3 can result in elevated extracellular glutamate, promoting hyperexcitability and neurotoxicity [67]. This heightened glutamatergic activity not only facilitates seizure propagation but also contributes to the pathophysiology of depression by inducing neuroinflammatory and neurodegenerative processes.

Overall, genetic predispositions that affect BBB permeability, seizure susceptibility, and glutamate sensitivity underscore the mechanistic link between epilepsy and depression. Understanding these genetic influences can provide insights into the conditions’ shared pathophysiological pathways and potentially guide the development of targeted therapeutic interventions.

## 8. Treatments for Brain–Blood Glutamate Equilibrium

BBB disruption determines and propagates the secretion of glutamate in epilepsy. The extent of the damage to the BBB affects its permeability and function. Addressing or preventing damage to the BBB may stop the progression of the positive feedback loop that promotes further excitotoxicity. We anticipate future research will focus more on the contribution of the BBB in epilepsy and potentially develop novel alternatives to current treatments that target BBB dysfunction.

We propose several interventions that can assist with preserving or restoring the integrity of the BBB (Table 1). The first method focuses on the structural integrity of the BBB. Targeting paracellular permeability could limit or reverse the leakage of substances between endothelial cells. Specifically targeting adherens junctions and tight junctions, as well as their regulators, might provide the biggest benefit given the pathology of the damaged BBB in epilepsy. Furthermore, inhibiting the expression of matrix metalloproteinases through TGF-β1 could also limit damage to the extracellular matrix [68,69].

The second approach aims to restore the functionality of the BBB, via the ATP-binding cassette (ABC) transporter. Potentiating the ABC would restore efflux transporter activity, which clears toxins from the brain. Furthermore, the repair of the neurovascular unit could also re-establish the normal function of neurons, astrocytes, endothelial cells, pericytes, and the basal lamina. It would ultimately restore microvascular blood flow, limit neuronal death, and promote neurogenesis and angiogenesis [70,71].

Lastly, targeting inflammation could address its downstream effects on BBB structure and function [72]. Specifically, COX-2 inhibition, AQP4 inhibition, the administration of docosahexaenoic acid (DHA), the inhibition of Na-K-Cl cotransporter, and the use of bone marrow mononuclear cells (MNCs) are potential targets in modulating inflammation [73,74].

## 9. Conclusions

This review elucidates the pathologies that arise with epilepsy, such as increased glutamate release and BBB dysfunction, and contribute to the emergence of neuropsychiatric disorders. We propose that epilepsy induces BBB dysfunction via hyperexcitation, triggering various forms of neurodegeneration. BBB dysfunction further contributes to hyperexcitation through a positive feedback loop. We examined factors influencing brain glutamate levels, such as neuronal cell death, inflammation, stress, and loss of synaptic integrity. Lastly, we listed potential therapeutic strategies that could target the BBB dysfunction seen in epilepsy. We anticipate that this review will be of great interest to the study of epilepsy and the therapeutic modalities aimed at preventing or reducing the condition’s comorbidities, especially those concerning mood and quality of life.

## Figures and Tables

**Table 1:** Treatments for brain–blood glutamate equilibrium.

Intervention	Methods/Targets	Expected Outcome
Structural integrity	Adherens junctions	Limit/reverse leakage between endothelial cells Limit damage to extracellular matrix
	Tight junctions Regulators of junctions Inhibition of matrix metalloproteinases via TGF-β1	
Restoring functionality	Potentiating ABC transporter	Restore efflux transporter activity
	Repairing neurovascular unit components (neurons, astrocytes, endothelial cells, pericytes, and basal lamina)	Re-establish normal function, restore microvascular blood flow, limit neuronal death, and promote neurogenesis and angiogenesis.
Targeting inflammation	COX-2 inhibition	Modulate inflammation and its effects on the BBB
	AQP4 inhibition	
	Administration of DHA	
	Inhibition of Na-K-Cl cotransporter	
	Use of bone marrow MNCs	

## Data Availability

No new data were created or analyzed in this study. Data sharing is not applicable to this article.
